# Government Actions and Their Relation to Resilience in Healthcare; What We See Is Not Always What We Get: Comment on "Government Actions and Their Relation to Resilience in Healthcare During the COVID-19 Pandemic in New South Wales, Australia and Ontario, Canada"

**DOI:** 10.34172/ijhpm.2021.170

**Published:** 2021-12-13

**Authors:** Ian P. Leistikow, Roland Bal

**Affiliations:** ^1^Dutch Health & Youth Care Inspectorate, Utrecht, The Netherlands.; ^2^Erasmus School of Health Policy & Management, Erasmus University, Rotterdam, The Netherlands.

**Keywords:** Resilience in Healthcare, Government Policy, Regulation, Healthcare Quality

## Abstract

This commentary reviews the publication by Smaggus et al published in the *IJHPM* in July 2021 on "Government Actions and Their Relation to Resilience in Healthcare During the COVID-19 Pandemic in New South Wales, Australia and Ontario, Canada" which analysed media releases to identify how governments contributed to resilience in healthcare (RiH). We suggest media releases might not be the best data to capture the mechanisms, activities and interactions through which government actions enhance or hinder RiH. RiH recognizes healthcare as a complex sociotechnical system, so studies into fostering capacity for RiH should be designed for complex sociotechnical systems. This means data should be derived from multiple sources to allow for diverse perspectives, and preferably include direct observations to capture the intricacies of backstage interactions.

 Smaggus and colleagues^1^ sought to investigate how governmental actions during the coronavirus disease 2019 (COVID-19) pandemic contributed to the potential for resilient performance in healthcare and what opportunities exist for governments to foster resilience within healthcare systems. The authors analysed media releases issued by two regional governments – New South Wales, Australia and Ontario, Canada – to identify themes relevant to the resilience potentials (anticipate, monitor, respond, learn) and resilience in healthcare (RiH). The paper contributes to the discussion how governments can help healthcare providers improve the resilience of their organisations and employees. This issue has become particularly pertinent during the COVID-19 pandemic but had been of interest already due to the increasing challenges healthcare faces. For example, in July 2019 National Health Service (NHS) England published their Patient Safety Strategy which strived to embed resilience principles (framed as ‘Safety-II’) in the national policy.^2^ Smaggus and colleagues draw attention to the scarcity of empirical investigations that examine how government actions contribute to the capacity for resilient performance in the healthcare setting and aim to address that gap with their study. In this commentary, we discuss the focus and method of their study, and suggest alternative paths for similar research. The two main issues we will raise are: (1) government and healthcare providers have distinct roles in relation to RiH and confusing these will cloud research results; (2) the frontstage performance and backstage conduct of governments are not always aligned, especially during crises. After discussing these issues, we will propose alternative ways to study the role of governments in fostering RiH.

 Our first comment relates to the distinct roles of government and healthcare providers in relation to RiH. RiH is defined as “the capacity to adapt to challenges and changes at different system levels, to maintain high quality care.”^3^ Governments provide the structure within which healthcare is provided, ie, the laws, regulations, financial and accountability structures. In countries using an NHS system, governments even own healthcare facilities. But governments do not treat patients. The actual delivery of high-quality care takes place on the interface between healthcare providers and those they provide healthcare to. Research into how government actions contribute to RiH should therefore not be aimed at resilient actions by governments, but at actions governments take to facilitate RiH delivery. In other words, how do governments help healthcare providers anticipate, monitor, respond and learn?

 In our own research, we have shown how governmental regulators can foster resilience within the sector they oversee by, for example, holding healthcare providers to account for how they empower their employees to be resilient and/or develop alternative ways of accounting for performance that help instead of hinder healthcare providers to stimulate resilience.^4^ As Smaggus wrote in an earlier article, success in complex systems requires front-line workers to adapt to dynamic circumstances and vary their behaviour to match their conditions.^5^ To give an example from the country we are most familiar with: at a webinar for medical staff on March 18, 2020, in the first weeks of the COVID-19 pandemic in the Netherlands, the director of the Dutch Health & Youth Care Inspectorate urged doctors to not let guidelines or regulations get in the way of treating their patients^[1]^. This created space for healthcare professionals to respond and adjust to the situation as it evolved and can be seen as a governmental action to increase RiH. The webinar where this statement was made was not publicly accessible, and specifically meant for medical doctors. This brings us to our second comment.

 Our second comment relates to using governmental media releases to understand how governments contribute to RiH. The director of the Inspectorate would most likely have phrased the statement we described above differently had it been for a media release. As the COVID-19 pandemic hit, and a large part of the population was disoriented and scared, it would not be fitting for government to issue a press release implying that the regulator was letting go of rules and guidelines, suggesting that professionals and hospitals were to put quality standards aside to take care of COVID-19 patients. The frontstage performance and backstage actions of governments are not always aligned, as they do not always serve the same purpose (a classic for the distinction between front- and back stage is Goffman^6^; see Bijker et al^7^ for an analysis of scientific advice using this perspective). Maintaining the public trust and a sense of control requires different strategies than getting things done, especially in times of uncertainty. Smaggus et al decided to use governmental media releases as their main data source. We however question whether media releases provide for realistic insight into government actions to enhance RiH during the COVID-19 pandemic as these would be typically used to convince the public that government is in control. It would have been informative to also look behind the scenes, not just at the government deliberations and differences in opinion as Smaggus et al suggest, but especially at how government interacted with healthcare providers to enhance the latter’s capacity for resilience. Including data from interviews and observations would have added an insightful layer to the research.

 We agree with Smaggus et al that developing an understanding of how governments foster (or compromise) the capacity for resilience within healthcare is a crucial task. Because RiH recognizes healthcare as a complex sociotechnical system, studies into fostering capacity for RiH should be designed for complex sociotechnical systems. This means data should be derived from different sources, to allow for diverse perspectives. And data should as far as possible be based on direct observations to capture Work as Done. This is especially important when data used for research is also data used for accountability, as those being held accountable have an interest in looking good. In pandemic times, such direct observations might of course be difficult, but not impossible. Given the position of the authors of the Smaggus et al paper they might have been able to contact ministries of health and healthcare organisations for (online) observations and interviews. In our work, we have followed decision-making in a large university hospital in the Netherlands, observing crisis management meetings, hygienic work, negotiations between regional and national crisis organisations and interviewing managers, policymakers, professionals, and patient representatives. Through this, we have for example focused on the work done at hospital level to cope with scarcity and uncertainty^8^ and the adaptive capacity of the Dutch crisis organization to deal with the different ‘waves’ of the pandemic.^9^ Our findings stipulate the importance of informal contacts between policymakers, regulators and healthcare organisations; apart from the webinar mentioned above, such contacts were many—either through online meetings, Whatsapp groups, telephone calls, or visits. Such informal – backstage – contacts cannot be found in official documentation and even in the regular media (who have played a large role in the public discussion on pandemic policymaking) it is difficult to find all the subtleties of communication patterns. Getting an ‘inside view’ then helps in understanding how governments help or hinder resilience at the ground floor.

 We think there is also room to move further theoretically. Much of the literature that Smaggus et al use comes from a health services research or safety science background and is predominantly focused on the organizational level. The insights this literature provides are relevant, but they are hardly enough for studying public policy, politics and administration. For example, whilst it is hard to get organizations moving, governments are even more resistant to change. Within public administration, the concept of path dependency illustrates how previous choices get institutionalized in regulations, organisations, financial arrangements, cultural and social norms.^10^ As a consequence, governmental bureaucracies are good at responding to known risks (they are designed to ‘win the last war’) but find it difficult to adapt to new threats. To illustrate; testing during the COVID-19 pandemic in the Netherlands was a scarce capacity. This had to do with the ways in which testing has been institutionalized in the past, with many small and local laboratories instead of fully automated large testing facilities that can be seen in eg, France and Germany. There is a good reason for this in the Netherlands; the country is proud of its low use of medicines, especially antibiotics, and the expertise-led testing labs play a large role in sustaining this policy. During pandemic times however, small scale testing does not work well. Dutch government has had to go through great lengths to develop a work-around for this, risking however that this opens up the testing market to the big companies that would then undermine prudent testing policies of the past. To get a grip on such effects as researcher, not only is it necessary to get acquainted with the intricacies of healthcare systems, but also to have an understanding of the possible perverse effects of policies, and of the institutional dynamics of public administration.

 Summing up, we underscore the importance of empirical research into how government actions contribute to the capacity for RiH. The international RiH research program suggests four questions that studies of RiH need to consider when operationalising resilience. These are: resilience ‘for what,’ ‘to what,’ ‘of what,’ and ‘through what’?^3^ To gain a better understanding of the mechanisms through which government actions enhance or hinder RiH, research should use concepts from the political and administrative science literatures and allow for multiple sources, preferably including direct observations to capture the intricacies of backstage interactions.

## Ethical issues

 Not applicable.

## Competing interests

 Authors declare that they have no competing interests.

## Authors’ contributions

 Both authors contributed equally to this work.

## Endnote

 [1] Note to editor: a summary of the webinar can be found at https://www.zwollenu.nl/wp-content/uploads/2020/03/FMS_Webinar_COVID-19_2020-03-18.x85913.pdf.
